# Optical coherence tomography angiography as a potential screening tool for cerebral small vessel diseases

**DOI:** 10.1186/s13195-020-00638-x

**Published:** 2020-06-11

**Authors:** Ju-Yeun Lee, Jun Pyo Kim, Hyemin Jang, Jaeho Kim, Sung Hoon Kang, Ji Sun Kim, Jongmin Lee, Young Hee Jung, Duk L. Na, Sang Won Seo, Sei Yeul Oh, Hee Jin Kim

**Affiliations:** 1 Department of Ophthalmology, Myongji Hospital, Hanyang University College of Medicine, Goyang, Republic of Korea; 2 Department of Neurology, Samsung Medical Center, Sungkyunkwan University School of Medicine, Seoul, Republic of Korea; 3 Samsung Alzheimer Research Center, Samsung Medical Center, Seoul, Republic of Korea; 4 Neuroscience Center, Samsung Medical Center, Seoul, Republic of Korea; 5 Department of Neurology, Myongji Hospital, Hanyang University College of Medicine, Goyang, Republic of Korea; 6 Department of Health Sciences and Technology, SAIHST, Sungkyunkwan University, Seoul, Republic of Korea; 7 Department of Clinical Research Design & Evaluation, SAIHST, Sungkyunkwan University, Seoul, Republic of Korea; 8 Department of Digital Health, SAIHST, Sungkyunkwan University, Seoul, Republic of Korea; 9 Department of Ophthalmology, Samsung Medical Center, Sungkyunkwan University School of Medicine, Seoul, Korea

**Keywords:** Optical coherence tomography angiography, Cerebral small vessel disease, Alzheimer’s disease, Subcortical vascular dementia

## Abstract

**Background:**

The retina and the brain share anatomic, embryologic, and physiologic characteristics. Therefore, retinal imaging in patients with brain disorders has been of significant interest. Using optical coherence tomography angiography (OCTA), a novel quantitative method of measuring retinal vasculature, we aimed to evaluate radial peripapillary capillary (RPC) network density and retinal nerve fiber layer (RNFL) thickness in cognitively impaired patients and determine their association with brain imaging markers.

**Methods:**

In this prospective cross-sectional study, a total of 69 patients (138 eyes) including 29 patients with amyloid-positive Alzheimer’s disease-related cognitive impairment (ADCI), 25 patients with subcortical vascular cognitive impairment (SVCI), and 15 amyloid-negative cognitively normal (CN) subjects were enrolled. After excluding eyes with an ophthalmologic disease or poor image quality, 117 eyes of 60 subjects were included in the final analyses. Retinal vascular [capillary density (CD) of the radial peripapillary capillary (RPC) network] and neurodegeneration markers [retinal nerve fiber layer (RNFL) thickness at four quadrants] were measured using OCTA and OCT imaging. Brain vascular (CSVD score) and neurodegeneration markers (cortical thickness) were assessed using 3D brain magnetic resonance imaging. The CD and RNFL thickness and their correlation with brain imaging markers were investigated.

**Results:**

The SVCI group showed lower CD in the temporal quadrant of the RPC network compared to the CN group (mean (SD), 42.34 (6.29) vs 48.45 (7.08); *p* = 0.001). When compared to the ADCI group, the SVCI showed lower CD in the superior quadrant (mean (SD), 60.14 (6.42) vs 64.15 (6.39); *p* = 0. 033) as well as in the temporal quadrant (ADCI 45.76, SVCI 42.34; *p* = 0.048) of the RPC network. The CD was negatively correlated with CSVD score in the superior (B (95%CI), − 0.059 (− 0.097 to − 0.021); *p* = 0.003) and temporal (B (95%CI), − 0.048 (− 0.080 to − 0.017); *p* = 0.003) quadrants of the RPC network. RNFL thickness did not differ among the groups nor did it correlate with cortical thickness.

**Conclusions and relevance:**

The microvasculature of the RPC network was related to the CSVD burden. However, the RNFL thickness did not reflect cerebral neurodegeneration. Noninvasive and rapid acquisition of the OCTA image might have the potential to be used as a screening tool to detect CSVD.

## Background

The most common causes of dementia are Alzheimer’s disease (AD) and cerebral small vessel disease (CSVD) [1]. In AD-related cognitive impairment (ADCI), amyloid-beta (Aβ) deposition and neurodegeneration are the hallmarks [2] whereas in subcortical vascular-related cognitive impairment (SVCI), CSVD markers such as lacunes, white matter hyperintensity (WMH), and microbleeds are the hallmarks [3]. To screen for neurodegeneration or CSVD, a simple, noninvasive, and inexpensive tool is necessary.

The retina and the brain share anatomic, embryologic, and physiologic characteristics. Since the retina might reflect the status of the brain condition, various studies on retinal imaging in dementia patients have been performed [4–25]. In terms of neurodegeneration, some studies suggested decreased retinal nerve fiber layer (RNFL) thickness in AD patients compared to controls [4–10], while other studies showed no significant changes [11–15, 26, 27]. Regarding microvascular alteration, some studies showed reduced vascularity in AD patients [9, 16, 20, 28], while a study showed increased vascularity in preclinical AD patients [15]. In the previous studies, however, the change in the vessels was manually assessed at the arteriolar level with classic morphometric methods which are difficult to quantify capillary perfusion. Also, most studies focused on parafoveal vasculature, and few studies evaluated the microvasculature of the peripapillary region (around the optic disc). We focused on the four quadrants of the peripapillary region since the optic disc is known to be susceptible to ischemic conditions, and there is a disease-specific regional vulnerability in the peripapillary area [29–31].

Optical coherence tomography (OCT) angiography (OCTA), recently introduced into clinical practice, is considered a noninvasive and novel technique to detect blood flow by acquiring the de-correlation signal between consecutive OCT cross-sectional scans repeated at the same location [32, 33]. In particular, swept-source (SS) OCTA provides a high 100-kHz A-line rate and deep signal penetration through the retina and choroid [32]. The retinal microvascular plexus can be visualized and segmented through a layer-by-layer analysis in this OCTA system [32]. Thus, OCTA has advantages over classic methods in that we can quantify the retinal microvasculature more accurately, and RNFL thickness can be measured simultaneously. Using OCTA images, some researchers tried to elucidate perfusion and architectural alternations in retinal microvasculature in patients with dementia [15, 34–37]. However, most of those studies were limited to patients with AD. In addition, although a previous study reported the relationship between the microvasculature and brain imaging markers [38], it has not been studied using the OCTA technique.

We prospectively recruited ADCI, SVCI, and controls. We aimed to compare capillary density (CD) of the radial peripapillary capillary (RPC) network and RNFL thickness between the groups using OCTA imaging. Then, we evaluated whether the CD in the RPC network correlated with CSVD markers and whether RNFL correlated with Aβ or cortical thickness. We hypothesized that the SVCI group would have the lowest RPC density while the AD group would show the lowest RNFL thickness among the three groups. We also hypothesized that CD in the RPC network would reflect CSVD burden, and RNFL thickness would reflect AD-related neurodegeneration.

## Methods

### Participants

**Fig. 1:**
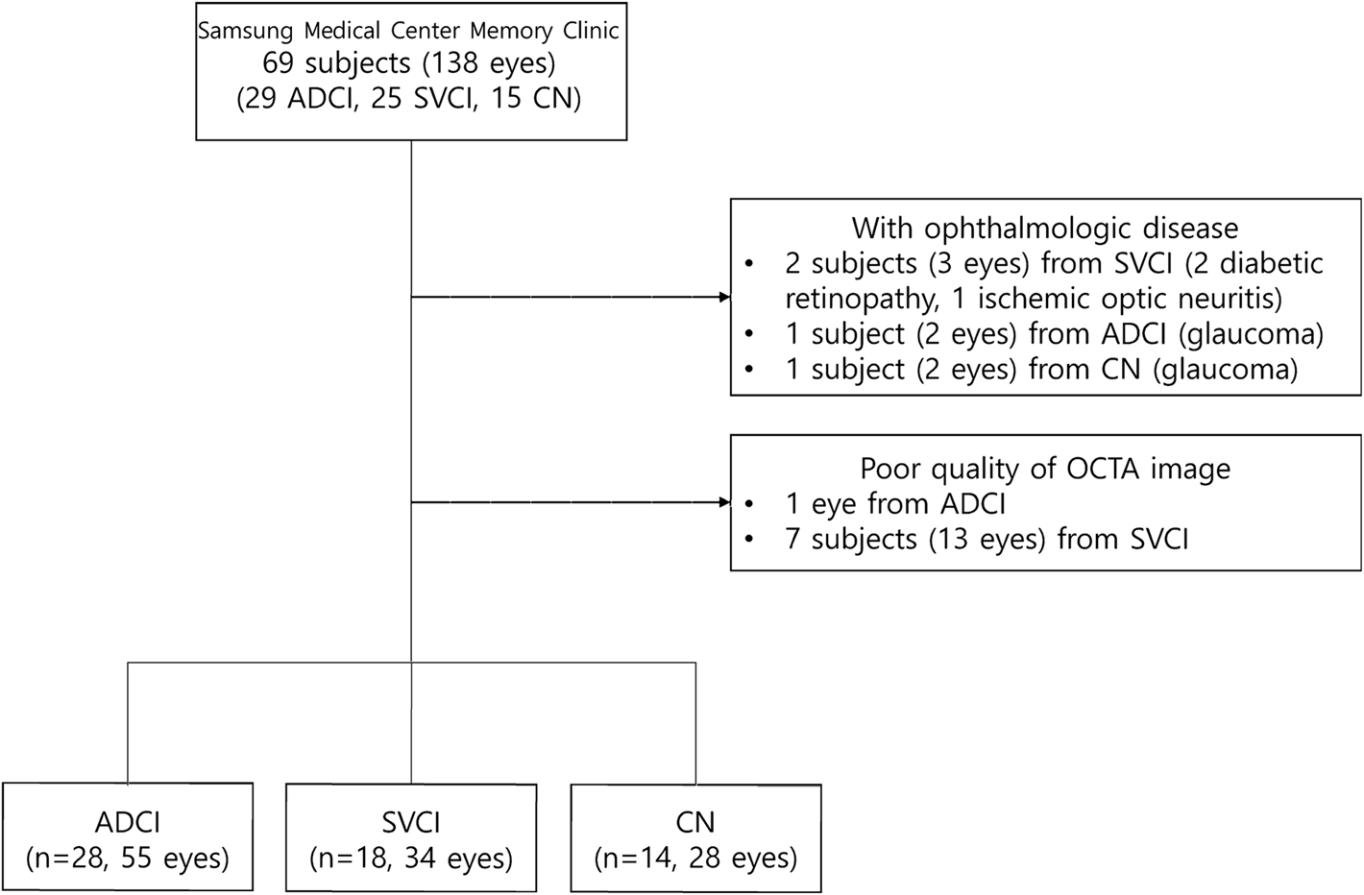
Flowchart for inclusion and exclusion of participants. Process of identifying final participants in Alzheimer’s disease-related cognitive impairment (ADCI), subcortical vascular cognitive impairment (SVCI), and cognitive normal (CN) groups

A total of 29 patients with ADCI, 25 patients with SVCI, and 15 normal controls were prospectively recruited from the memory clinic at Samsung Medical Center, Seoul, Korea, from February 2018 to March 2019 (Fig. 1). Based on a previous meta-analysis study which showed an effect size of 0.98 for the difference in mean RNFL thickness between AD and controls [39], we estimated that a sample size of 18 (number of eyes) for each group achieves 80% power to detect the difference of RNFL thickness between ADCI and healthy controls with a significance level (*α*) of 0.05. Since OCTA is a recently developed tool to measure CD of the RPC network, no previous literature was available to estimate the sample size for the SVCI group.

ADCI comprises AD dementia and amnestic mild cognitive impairment (aMCI) due to AD, which was diagnosed based on the National Institute on Aging–Alzheimer’s Association research criteria for probable AD dementia and aMCI due to AD, respectively [40]. These patients showed objective cognitive impairment less than the 16th percentile of the age- and education-matched norm on neuropsychological tests [41]. The presence of amyloid burden was confirmed by amyloid positron emission tomography (PET). We excluded patients with severe WMH, defined as periventricular WMH over 10 mm and deep WMH over 25 mm on brain magnetic resonance imaging (MRI).

SVCI was diagnosed based on the following criteria [42–44]: (1) subjective cognitive complaint by the patient or caregiver; (2) objective cognitive impairment less than the 16th percentile of the age- and education-matched norm in any domain including language, visuospatial, memory, or frontal function on neuropsychological tests [41]; (3) presence of severe ischemia on brain MRI; and (4) focal neurologic symptoms or signs.

Cognitively normal (CN) was defined by the following criteria: (1) no history of neurologic or psychiatric disorders, (2) normal cognitive function determined using neuropsychological tests (greater than 16th percentile of the age- and education-matched norm) [41], (3) absence of amyloid burden confirmed by amyloid PET, and (4) absence of severe WMH on brain MRI. Blood tests to exclude secondary causes of dementia included complete blood cell count, blood chemistry, vitamin B12/folate analysis, syphilis serology, thyroid function tests, and apolipoprotein E genotyping. We excluded participants who showed structural lesions, including territorial cerebral infarction, cortical stroke, brain tumor, hippocampal sclerosis, or vascular malformation on brain MRI. Participants who had concurrent retinal diseases (e.g., diabetic neuropathy, epiretinal membrane, or macular degeneration), any history of glaucoma, optic neuropathies, and ocular surgery except for cataract surgery were also excluded.

### OCTA image acquisition and analysis

Retinal peripapillary microvasculature was analyzed using a Topcon OCT instrument (DRI OCT Triton plus) for all patients and healthy controls. The Triton swept-source OCT uses a wavelength of 1050 nm with a scan speed of 100,000 A-scans per second. The instrument uses an active eye tracker that follows eye movement; it detects blinking and adjusts the scan position accordingly, thereby reducing the motion artifact during OCTA image generation. Each patient underwent imaging consisting of a 4.5 × 4.5 mm diameter peripapillary scan centered on the optic disc. Scans with poor quality, defined by the following criteria, were not included: (1) image quality score < 40 [45], (2) poor clarity, (3) residual motion artifacts visible as an irregular vessel pattern on the en-face angiogram, (4) local weak signal, and (5) off-centered optic disc. The RPC networks were separated automatically through layer segmentation with the OCT instrument software (IMAGEnet 6 V.1.14.8538). The RPC extended from 3 μm below the internal limiting membrane (ILM) to 15 μm below the inner plexiform layer (IPL). The RPC network segment extended from the ILM to the posterior boundary of the RNFL. Quantitative values of CD in the RPC network were defined as the percentage of the area occupied by the vessels in a localized region. The software automatically fitted an Early Treatment Diabetic Retinopathy Study (ETDRS) inner circle to the optic disc [46, 47]. Four areas (nasal, temporal, superior, and inferior) dividing the center on the disc are automatically displayed. The CD of each area is indicated as a percentage.

### MRI acquisition and measurement of cortical thickness

All subjects underwent a 3D volumetric brain MRI scan. An Achieva 3.0-Tesla MRI scanner (Philips, Best, the Netherlands) was used to acquire 3D T1 Turbo Field Echo (TFE) MRI data using the following imaging parameters: sagittal slice thickness, 1.0 mm with 50% overlap; no gap; repetition time of 9.9 ms; echo time of 4.6 ms; flip angle of 8°; and matrix size of 240 × 240 pixels reconstructed to 480 × 480 over a field of view of 240 mm.

For cortical thickness measurements, T1-weighted MRIs were automatically processed using the standard Montreal Neurological Institute image processing software (CIVET). This software has been well-validated and is extensively described elsewhere, including in aging/atrophied brain studies [48, 49].

### Assessment of lacunes, WMH, and microbleeds on brain MRI

Lacunes were defined as lesions (≥ 3 mm and ≤ 15 mm in diameter) with low signal on T1-weighted images, high signal on T2-weighted images, and a perilesional halo on the axial sections of fluid-attenuated inversion recovery (FLAIR) images. We quantified WMH volume (in milliliters) on FLAIR images using an automated method. Microbleeds were defined as 10 mm or less in diameter, using the criteria proposed by Greenberg et al. [50] on 20 axial sections of the time constant for T2*-weighted gradient-recalled echo sequence MRIs. Detailed measurement methods for lacunes and microbleeds were described previously [42]. Using the numbers of lacunes and microbleeds and the WMH volume, we defined a CSVD score based on the method used in our previous study [44]. The CSVD score (0–3) awards 1 point each for lacune (if present), microbleed (if present), and WMH volume (if greater than the median value of 3.82 mL).

### PET acquisition and interpretation

We used ^18^F-florbetaben PET or ^18^F-flutemetamol PET to detect amyloid in the brain. PET images were rated as amyloid positive or negative by a nuclear medicine physician. ^18^F-florbetaben PET was defined as positive when visual assessment scored 2 or 3 on the brain Aβ plaque load (BAPL) scoring system [51]. Visual interpretation of ^18^F-flutemetamol PET images relied upon a systematic review of five brain regions (frontal, parietal, posterior cingulate and precuneus, striatum, and lateral temporal areas). If any of the brain regions were positive in either hemisphere, the scan was considered positive [52].

### Statistical analysis

For comparison of clinical characteristics, the Mann-Whitney test for continuous variables and the chi-square test or Fisher’s exact test for categorical variables were used. Regarding radiological parameters such as CSVD markers and cortical thickness, we performed the analysis of covariate (ANCOVA) with Bonferroni corrections for multiple comparisons, controlling for age, sex, hypertension, and diabetes. For WMH volume and cortical thickness, we additionally controlled for total intracranial volume.

Generalized estimation equation (GEE) models, accounting for age, sex, hypertension, diabetes, within-patient intereye correlations, and image quality score [53], were used to compare the CDs between the groups with Bonferroni correction.

To evaluate the linear associations between CD in the RPC network and CSVD markers, we used the linear models accounting for age, sex, hypertension, diabetes, and image quality score. To assess the association between RNFL thickness and amyloid positivity, we performed logistic regression analyses accounting for age, sex, hypertension, diabetes, and image quality score. To evaluate the linear associations between RNFL thickness and cortical thickness, we used the linear models accounting for age, sex, hypertension, diabetes, image quality score, and intracranial volume. In the analyses, the CD and RNFL thicknesses from the randomly selected eye were included as independent variables. A *p* value of less than 0.05 was considered statistically significant. All continuous data were presented as mean ± standard deviation (SD). Statistical analyses were performed using R (version 3.5.3).

## Results

### Clinical and radiological characteristics

Among the 69 subjects (138 eyes) enrolled, three eyes from two SVCI subjects, two eyes from an ADCI subject, and two eyes from a CN subject were excluded for having ophthalmologic diseases. Also, 13 eyes from seven subjects in the SVCI group and one eye from a subject from the CN group were excluded from the statistical analysis for poor image quality due to motion artifacts. Therefore, 28 subjects (55 eyes) in the ADCI group, 18 subjects (34 eyes) in the SVCI group, and 14 subjects (28 eyes) in the CN group were finally included in the analysis. The mean age in the SVCI group was 77 years, which was higher than that in either the ADCI group (67.5 years, *p* = 0.004) or the CN group (67.2 years, *p* = 0.002). The mean year of education was higher in the ADCI group (11.9 years) compared to the CN group (7.9 years, *p* = 0.036). The mean K-MMSE score was higher in the CN group (28.0) compared to the ADCI (20.6, *p* < 0.001) or the SVCI group (21.5, *p* < 0.001). The proportion of *Apolipoprotein E* e4 carriers in the ADCI group (60.7%) was significantly higher than that in the SVCI group (22.2%, *p* = 0.046) and the CN group (14.3%, *p* = 0.011). There was no difference in sex, hypertension, and diabetes between the groups.

In terms of CSVD markers, subjects in the SVCI group had higher CSVD scores (ADCI 0.6, SVCI 2.1, CN 0.5) and WMH volumes (ADCI 5.2 mL, SVCI 45.9 mL, CN 4.2 mL) compared with the ADCI or CN group (*p* < 0.001 for all comparisons). Cortical thickness was significantly lower in the ADCI group compared with the CN group regardless of anatomical regions, while there was no significant difference between the SVCI and CN groups. Cortical thickness was lower in the ADCI group than in the SVCI group (2.95 mm vs 3.03 mm, *p* < 0.001). Clinical and radiological characteristics in all groups are summarized in Table 1.

**Table 1:** Clinical and radiological characteristics of participants

	ADCI, *n* = 28, 55 eyes	SVCI, *n* = 18, 34 eyes	CN, *n* = 14, 28 eyes	*p* values		
				ADCI vs CN	SVCI vs CN	ADCI vs SVCI
Age, years	67.5 (9.5)	77.0 (6.3)	67.2 (6.1)	1.000	0.002	0.004
Sex (M/F)	11/17	6/12	4/10	1.000	1.000	1.000
Education, years	11.9 (4.2)	8.9 (5.9)	7.9 (4.3)	0.036	1.000	0.315
K-MMSE	20.6 (5.1)	21.5 (4.6)	28.0 (1.9)	< 0.001	< 0.001	1.000
Amyloid positivity, no. (%)	28 (100%)	6 (33%)	0 (0%)	< 0.001	0.071	< 0.001
*APOE e4* carrier, no. (%)	17 (61%)	4 (22%)	2 (14%)	0.023	1.000	0.046
Hypertension, no. (%)	12 (43%)	11 (61%)	9 (64%)	0.570	1.000	0.681
Diabetes, no. (%)	3 (11%)	5 (28%)	4 (29%)	0.591	1.000	0.696
CSVD score	0.6 (0.8)	2.1 (0.6)	0.5 (0.9)	1.000	< 0.001	< 0.001
Number of lacunes	0.1 (0.4)	1.7 (3.3)	0.3 (0.8)	1.000	0.578	0.284
Number of microbleeds	1.4 (5.2)	6.6 (15.9)	0.1 (0.3)	1.000	0.273	0.305
WMH volume, mL	5.2 (8.4)	45.9 (19.8)	4.2 (4.8)	1.000	< 0.001	< 0.001
Cortical thickness, mm	2.95 (0.17)	3.03 (0.16)	3.14 (0.07)	< 0.001	0.463	0.113

*ADCI* Alzheimer’s disease cognitive impairment, *SVCI* subcortical vascular cognitive impairment, *CN* cognitively normal, *K-MMSE* Korean version of mini-mental status examination, *WMH* white matter hyperintensity

### Comparisons of retinal microvasculature and RNFL thickness in the ADCI, SVCI, and CN groups

**Fig. 2:**
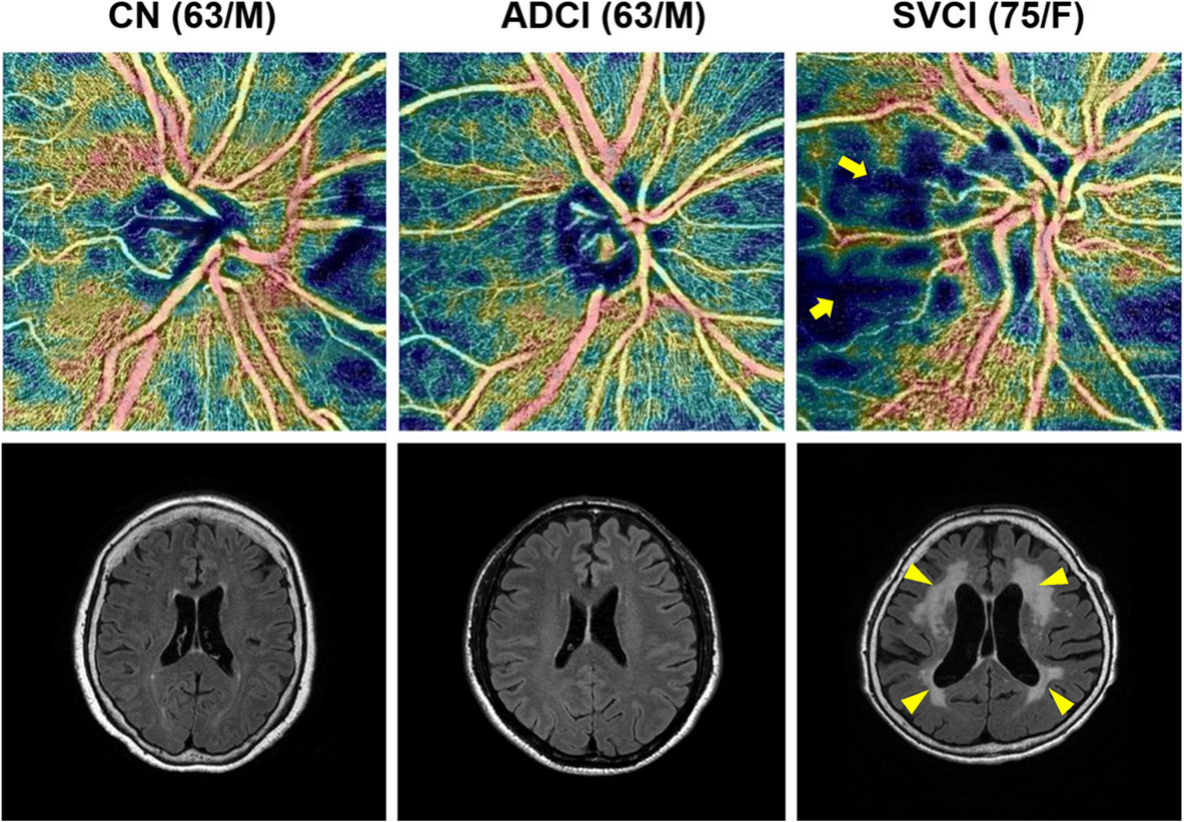
Representative images according to diagnostic groups. Representative patient images of optical coherence tomography angiography and brain magnetic resonance imaging. Images of the superficial radial peripapillary capillary network (upper row) and axial T2 fluid-attenuated inversion recovery (lower row) of cognitively normal (CN), Alzheimer’s disease-related cognitive impairment (ADCI), and subcortical vascular cognitive impairment (SVCI) subjects. The SVCI patient shows decreased peripapillary capillary network density in the temporal quadrant (arrows) and severe subcortical white matter hyperintensity (arrowheads)

With respect to the RPC network, the CD of the SVCI group was significantly lower than that of the CN group in the temporal quadrant (SVCI 42.34, CN 48.45; *p* = 0.001; Cohen’s *d* = 0.915). Also, the CD in the RPC network was significantly lower in the SVCI group as compared with the ADCI group in the superior quadrant (ADCI 64.15, SVCI 60.14, *p* = 0.039; Cohen’s *d* = 0.625) as well as in the temporal quadrant (ADCI 45.76, SVCI 42.34, *p* = 0.048; Cohen’s *d* = 0.510). Representative images of the RPC network and WMH in each group are shown in Fig. 2. There was no significant difference in RNFL thickness between the groups regardless of the region. Notably, RNFL and CD in the RPC network of the ADCI group were not significantly different from those of the CN group. These negative findings are supported by their lower than medium effect size (Cohen’s *d* < 0.5). Detailed results are presented in Table 2.

**Table 2:** Comparisons of capillary density in the radial peripapillary capillary (RPC) network and retinal nerve fiber layer (RNFL) thickness among the three groups

	ADCI, *n* = 28	SVCI, *n* = 18	CN, *n* = 14	*p* values*		
				ADCI vs CN	SVCI vs CN	ADCI vs SVCI
Capillary density in the RPC network (%)						
Superior	64.15 (6.39)	60.14 (6.42)	63.16 (6.18)	1.000	0.121	**0.033**
Inferior	67.19 (7.34)	64.06 (6.07)	63.43 (7.8)	0.238	1.000	0.171
Temporal	45.76 (7.13)	42.34 (6.29)	48.45 (7.08)	0.471	**0.001**	**0.048**
Nasal	49.69 (5.52)	50.25 (6.29)	50.51 (5.59)	1.000	1.000	1.000
RNFL thickness (μm)						
Superior	129.80 (19.2)	124.19 (21.73)	126.5 (16.44)	1.000	1.000	1.000
Inferior	138.25 (22.21)	128.51 (19.5)	138.1 (18.51)	1.000	1.000	1.000
Temporal	80.48 (12.13)	76.84 (15.36)	77.79 (10.83)	1.000	0.906	1.000
Nasal	76.33 (15.64)	78.73 (11.84)	81.57 (10.99)	0.404	1.000	0.740

Generalized estimation equation models after controlling for age, sex, hypertension, diabetes, and image quality score

*ADCI* Alzheimer’s disease cognitive impairment, *SVCI* subcortical vascular cognitive impairment, *CN* cognitively normal

*p values: after Bonferroni correction for multiple group comparison

### Association between the CD in the RPC network and CSVD

On the linear regression analysis among the entire subjects, the CDs were negatively associated with the CSVD score in the superior (B (95%CI), − 0.059 (− 0.097 to − 0.021); *p* = 0.003) and temporal (B (95%CI), − 0.048 (− 0.080 to − 0.017); *p* = 0.003) quadrants in the RPC network (Table 3). Then, we further performed linear regression analyses to find which components of the CSVD score correlated with CD in the superior and temporal quadrants of the RPC network among the entire subjects after false discovery rate correction. We found that the CD at the temporal quadrant was negatively associated with WMH volume (B (95%CI), − 0.966 (− 1.718 to − 0.214); *p* = 0.039).

**Table 3:** Association between the radial peripapillary capillary (RPC) network density and cerebral small vessel disease (*N* = 60)

Quadrants	CSVD score	
	B (95%CI)	*p*
Superior	− 0.059 (− 0.097 to − 0.021)	**0.003**
Inferior	− 0.026 (− 0.055 to 0.004)	0.085
Temporal	− 0.048 (− 0.080 to − 0.017)	**0.003**
Nasal	− 0.028 (− 0.066 to 0.011)	0.158

Linear regression models, after controlling for age, sex, hypertension, diabetes, and image quality score

*CSVD* cerebral small vessel disease, *CI* confidence interval

### Association between RNFL thickness and AD-related brain imaging markers

There was no significant association between RNFL thickness and amyloid positivity or between RNFL thickness and brain cortical thickness (Table 4).

**Table 4:** Association between the retinal nerve fiber layer (RNFL) thickness and AD-related imaging biomarkers (*N* = 60)

Quadrants	Amyloid positivity*		Cortical thickness, mm**	
	B (95%CI)	*p*	B (95%CI)	*p*
RNFL thickness, μm (× 10^3^)				
Superior	21.0 (− 17.2 to 62.3)	0.289	0.917 (− 2.425 to 4.258)	0.580
Inferior	− 10.1 (− 48.5 to 25.8)	0.586	− 0.529 (− 3.649 to 2.591)	0.732
Nasal	21.7 (− 39.1 to 83.4)	0.471	1.735 (− 3.185 to 6.654)	0.478
Temporal	− 1.1 (− 51.3 to 49.5)	0.965	2.168 (− 2.019 to 6.355)	0.299

*RNFL* retinal nerve fiber layer, *CI* confidence interval

*Logistic regression models after controlling for age, sex, hypertension, diabetes, and image quality score

**Linear regression models after controlling for age, sex, hypertension, diabetes, image quality score, and intracranial volume

## Discussion

In the current study, we examined CD in the RPC network and RNFL using SS-OCTA, a relatively novel method in well-characterized patients with ADCI, SVCI, and CN individuals, and evaluated whether those measurements were correlated with imaging markers. We found that the CD in the RPC network was significantly lower in the SVCI group compared to the CN or ADCI group, showing a medium to large effect size. Also, the CD in the RPC network had a significant association with the CSVD burden in terms of the WMH volume. On the other hand, RNFL thickness was not different between the groups, nor did it correlate with amyloid positivity or cerebral cortical thickness. Taken together, we suggest that the OCTA might be a potentially useful tool to predict CSVD.

Our first major finding was that the RPC network densities in the SVCI group were lower than those in the ADCI or CN group. This result is in line with previous studies showing worse retinal vascular parameters in subjects with cerebral vascular changes: Retinal vascular parameters such as arterial narrowing, sclerosis [38, 54], and decreased arteriolar fractal dimension [18, 20] were reported to be associated with CSVD. However, these previous studies used manually measured retinal vasculature, which might have resulted in low clinical uses. Using SS-OCTA, we could easily examine the microvasculature down to the capillary level. While fluorescein angiography might be used for a similar purpose, the SS-OCTA is much simpler as it does not require intravenous dye injection and also provides more precise spatial information.

Of note, there was a regional vulnerability of decreased CD in the RPC network. CD in the temporal quadrant of the RPC network was significantly decreased in the SVCI group compared to the ADCI or CN group. The underlying pathophysiology of our findings might be related to vascular supply in the peripapillary region. The majority (60%) of the watershed zones lie in the temporal half of the optic nerve (RPC network). It is thus relatively less perfused during hemodynamic stress, making it more vulnerable to ischemic insult [55]. Since the temporal quadrant of the papillomacular bundle is related to the central visual field and visual acuity, microvascular loss in this area might also be clinically important. In addition, CD in the superior quadrant of the RPC network was decreased in the SVCI group compared to the ADCI group. Differential mechanical forces and regional anatomic differences at the level of the lamina cribrosa may account for varied susceptibility, particularly in the superior quadrant. We suggest the possibility that CD in the RPC network, particularly in the temporal and superior quadrant, might have a role as a screening biomarker of CSVD.

Our data showed that there was no difference in CD in the RPC network between the ADCI and CN groups. Results about peripapillary vascularity in AD patients varied in previous studies. Several previous studies [9, 28, 35] showed decreased peripapillary vascularity in AD patients, while others showed no difference [22] or even increase in peripapillary vascularity among preclinical AD patients compared to normal controls [15]. Such seemingly contradictory findings could be a consequence of the heterogeneity of AD participants in each study. AD participants of each study differ in the presence of AD biomarkers, the degree of CSVD burden combined, or the phase of the disease. We included amyloid biomarker-confirmed ADCI patients according to recently revised research criteria [40, 56], and excluded patients with severe WMH. It is possible that, in previous studies, decreased peripapillary vascularity in AD might have been driven by CSVD such as WMH. In the early phase of AD (preclinical AD), amyloid-related inflammation might increase the blood flow as a compensatory mechanism [57] which might be reversed in the late phase. Further studies with a larger sample size of homogenous AD patients are required to verify our results.

Our second major finding was that the CD in the RPC network was negatively associated with the CSVD burden especially in the superior and temporal quadrant. Based on the possible susceptibility to ischemic insults, those regions of the optic disc may well capture ischemic changes in the brain. Previous studies have shown that retinal vasculopathy was significantly associated with cerebral microbleeds [58], lacunar infarction [21], and white matter hyperintensity [16]. A more recent study demonstrated a significant negative association between cerebral WMH volume and arteriolar fractal dimension of retinal vasculature [18]. In these studies, arteriovenous nicking, microaneurysms/hemorrhage, arteriolar/venular caliber, and arteriolar fractal dimension were used as retinal vascular markers. Along with those markers, we found other evidence to support a significant relationship between the peripapillary architectural changes and the brain condition. Based on the results of this study, quantified microvasculature of the optic nerve might serve as one of the surrogate markers of CSVD with possible use in clinics.

Our last major finding was that RNFL thicknesses did not show any significant difference between the groups nor did they correlate with amyloid positivity or quantitative values of cerebral degeneration. Several studies reported thinner RNFL in AD or MCI patients compared to CN subjects [4–7, 59]. However, there were also some reports in which no significant difference in RNFL thickness between the AD and control groups was observed [11, 12, 15, 24, 39, 60, 61]. A recent prospective study with a large sample number showed no significant differences in the overall or regional RNFL thickness between the AD, MCI, and control groups [14]. Therefore, the relationship between peripapillary RNFL and neurodegenerative diseases remains somewhat controversial, and such discrepancies might suggest a lack of robust RNFL change in AD.

Our current study has the following limitations. First, although we prospectively recruited well-characterized subjects, this was a cross-sectional study with a relatively small sample size. Further longitudinal studies with a larger sample size are needed to confirm our results. Second, several SVCI patients were excluded due to poor image quality mainly caused by poor cooperation. Third, although the method we used to obtain the CSVD score was used in a previous study [44], it is not yet widely used. Also, since our SVCI patients already had severe CSVD, the value of OCTA markers as an early diagnostic tool remains to be determined. Further longitudinal studies that include cognitively unimpaired subjects at baseline are needed to verify this issue.

## Conclusion

In conclusion, this is the first study to comprehensively investigate CD in the RPC network and RNFL thickness in the ADCI and SVCI groups and their correlation with brain imaging markers. We showed that there are concomitant peripapillary and cerebral microvasculature changes, particularly in SVCI patients. Retinal neurodegeneration, as measured by peripapillary RNFL, was not significantly correlated with neurodegeneration of the brain, whereas peripapillary microvasculature might reflect the CSVD. Noninvasive and rapid acquisition of OCTA images has the potential to be used as a peripheral imaging tool to screen for the degree of CSVD.

## Data Availability

The datasets used and/or analyzed during the current study are available from the corresponding authors on reasonable request.

## Abbreviations

AD: Alzheimer’s disease
CSVD: Cerebral small vessel disease
ADCI: Alzheimer’s disease-related cognitive impairment
Aβ: Amyloid-beta
SVCI: Subcortical vascular-related cognitive impairment
WMH: White matter hyperintensity
RNFL: Retinal nerve fiber layer
OCT: Optical coherence tomography
OCTA: Optical coherence tomography angiography
SS-OCTA: Swept source optical coherence tomography angiography
RPC: Radial papillary capillary
aMCI: Amnestic mild cognitive impairment
PET: Positron emission tomography
MRI: Magnetic resonance imaging
CN: Cognitively normal
ILM: Internal limiting membrane
CD: Capillary density
ETDRS: Early Treatment Diabetic Retinopathy Study
TFE: Turbo Field Echo
FLAIR: Fluid-attenuated inversion recovery
BAPL: Brain Aβ plaque load
ANCOVA: Analysis of covariate
GEE: Generalized estimation equation
SD: Standard deviation
MMSE: Mini-Mental Status Examination
